# Probable everolimus-associated severe colitis with hematochezia in a patient with metastatic hormone receptor–positive, HER2-negative breast cancer: a case report

**DOI:** 10.3389/fphar.2026.1871050

**Published:** 2026-09-15

**Authors:** Ahmad Fatayer, Salem K. Qupp, Joyce Morcos, ZainEdeen Zyadah, Alaa Alresheq, Jahed Bushnaq, Marwan Qubaja, Ola Abu Laban

**Affiliations:** 1 Department of Internal Medicine, Palestine Medical Complex, Ramallah, Palestine; 2 Faculty of Medicine, Al-Quds University, Jerusalem, Palestine; 3 Internal Medicine, Washington University of Health and Science, San Pedro, Belize; 4 Anatomical Pathology Department, Augusta Victoria Hospital, Jerusalem, Palestine; 5 Cancer Care Center, Augusta Victoria Hospital, Jerusalem, Palestine

**Keywords:** drug-induced colitis, everolimus, gastrointestinal toxicity, hormone receptor-positive breast cancer, metastatic breast cancer, mTOR inhibitor

## Abstract

**Background:**

Everolimus, an inhibitor of the mechanistic target of rapamycin (mTOR), is used with endocrine therapy for endocrine-resistant hormone receptor–positive, human epidermal growth factor receptor 2 (HER2)–negative advanced breast cancer. Although diarrhea is a recognized adverse effect, severe inflammatory colitis accompanied by gastrointestinal bleeding is rare and remains insufficiently characterized.

**Case Presentation:**

A 60-year-old woman with metastatic estrogen receptor–positive, progesterone receptor–positive, HER2-negative breast cancer involving the bone and liver presented with a 2-day history of severe abdominal pain, bloody diarrhea, and hematochezia approximately 6 months after starting exemestane, everolimus, and zoledronic acid. Contrast-enhanced computed tomography demonstrated marked circumferential wall thickening and submucosal edema involving predominantly the cecum, ascending colon, and proximal transverse colon, with pericolic fat stranding and mild free fluid. Colonoscopy showed diffuse mucosal erythema and edema, and colonic biopsy demonstrated moderate chronic active non-specific colitis with focal surface epithelial erosion and preserved mucosal architecture. No viral cytopathic changes or evidence of inflammatory bowel disease, dysplasia, or malignancy were identified. Cytomegalovirus testing of colonic tissue and peripheral blood was negative, and available stool investigations identified no infectious etiology. Everolimus was permanently discontinued. Management included bowel rest, intravenous fluids, initial antibiotics, intravenous methylprednisolone, transfusion of two units of packed red blood cells for symptomatic anemia, and nutritional support. Follow-up computed tomography demonstrated complete resolution of the colonic inflammatory abnormalities.

**Discussion:**

The diagnosis rested on the temporal relationship with everolimus exposure, multimodal objective evidence of colitis, structured assessment of competing diagnoses, and sustained recovery following permanent drug withdrawal and medical management. The event was retrospectively classified as Common Terminology Criteria for Adverse Events Grade 3 colitis, and a Naranjo score of five supported a probable adverse drug reaction. Histopathology confirmed active colitis but was non-specific and did not independently establish everolimus causality.

**Conclusion:**

Probable everolimus-associated severe colitis should be considered when patients receiving mTOR inhibitors develop acute abdominal pain, diarrhea, or gastrointestinal bleeding. Because histopathological findings may be non-specific, diagnosis requires integration of drug exposure, objective evidence of intestinal inflammation, careful exclusion of competing etiologies, and clinical evolution following withdrawal of the suspected agent.

## 1 Introduction

The mechanistic target of rapamycin (mTOR) is a serine/threonine kinase that regulates cellular growth, proliferation, metabolism, survival, protein synthesis, and angiogenic signaling. Dysregulation of the phosphoinositide 3-kinase/protein kinase B/mTOR pathway contributes to endocrine resistance in hormone receptor–positive breast cancer and provides the rationale for therapeutic mTOR inhibition. Everolimus, an oral mTOR inhibitor, is used with endocrine therapy in selected patients with hormone receptor–positive, HER2-negative advanced breast cancer; in the pivotal BOLERO-2 study, adding everolimus to exemestane significantly improved progression-free survival compared with exemestane alone (Baselga et al., 2011).

The recognized adverse-event profile of everolimus includes stomatitis, rash, fatigue, diarrhea, metabolic abnormalities, infections, and non-infectious pneumonitis (Rugo et al., 2014; Raphael et al., 2020). Although diarrhea is relatively common, severe inflammatory gastrointestinal injury is uncommon. Published reports describe a spectrum of serious gastrointestinal complications associated with everolimus, including hemorrhagic colitis, severe gastrointestinal hemorrhage, intestinal ulceration, and bowel perforation (Arakawa et al., 2022; Tsunematsu et al., 2019; Abe et al., 2023; Song et al., 2020). Enteritis has also been reported with both everolimus and temsirolimus, suggesting that clinically significant inflammatory intestinal injury may represent a rare toxicity of mTOR pathway inhibition rather than an effect exclusive to a single agent (Parithivel et al., 2011).

Diagnosing drug-associated colitis is challenging because clinical, radiological, endoscopic, and histopathological findings may be non-specific. Causality therefore requires consideration of the temporal relationship with drug exposure, objective evidence of intestinal inflammation, competing diagnoses, dechallenge response, and, where appropriate, formal adverse drug reaction assessment. This difficulty is compounded in the patient receiving systemic anticancer therapy, in whom infection, neutropenic enterocolitis, ischemia, immune-mediated colitis, radiation injury, anticoagulation-associated bleeding and direct malignant involvement may coexist or mimic one another, and in whom misattribution carries substantial therapeutic consequences, whether unnecessary laparotomy on the one hand or continued exposure to a causative agent on the other. We report a case of probable everolimus-associated severe colitis in a patient with metastatic hormone receptor–positive, HER2-negative breast cancer who presented with severe abdominal pain, bloody diarrhea, and hematochezia. The case is supported by radiological, endoscopic, histopathological, molecular, and clinical findings and is presented with a structured differential diagnosis, Common Terminology Criteria for Adverse Events (CTCAE) severity assessment, Naranjo causality assessment, clinical timeline, and explicit discussion of limitations.

## 2 Case presentation

### 2.1 Patient information and oncologic history

A 60-year-old woman with metastatic estrogen receptor–positive, progesterone receptor–positive, HER2-negative breast cancer involving the bone and liver presented in August 2025 with an acute 2-day history of bloody diarrhea, hematochezia, and severe diffuse abdominal pain radiating to the back.

Her oncologic history began in 2021 with a diagnosis of metastatic breast cancer involving the bone and liver. She initially received three cycles of docetaxel, doxorubicin, and cyclophosphamide (TAC), followed by letrozole, palbociclib, and zoledronic acid for approximately 4 years with good tolerance. In January 2025, positron emission tomography–computed tomography (PET-CT) demonstrated disease progression; cancer antigen 15-3 (CA 15-3) rose from 300 to 695 U/mL and carcinoembryonic antigen (CEA) from 15 to 33 ng/mL. She received palliative radiotherapy to the sacral spine in February 2025, and treatment was changed to exemestane, everolimus, and zoledronic acid the same month; follow-up PET-CT in July 2025 demonstrated a partial response.

Approximately 6 months after starting the everolimus-containing regimen, she developed acute severe abdominal pain with bloody diarrhea and hematochezia. She had a history of deep venous thrombosis and was receiving apixaban, which was withheld during the acute gastrointestinal bleeding episode. She had received no immune checkpoint inhibitor at any point in her treatment course, no non-steroidal anti-inflammatory drugs, and no antibiotics in the preceding 3 months.

### 2.2 Acute presentation and initial assessment

At admission the patient was hemodynamically stable, with an Eastern Cooperative Oncology Group (ECOG) performance status of 2, and was conscious, alert, and oriented. Abdominal examination showed diffuse tenderness with a soft abdomen and no guarding or rigidity. Initial laboratory evaluation demonstrated hypokalemia, which was corrected; serum amylase and lipase were repeatedly within reference ranges, serum lactate was within the reference range, and the patient was not neutropenic.

The available infectious evaluation included routine stool analysis, stool culture sampling, and *C. difficile* toxin testing; routine stool studies and *Clostridioides difficile* testing did not identify an infectious cause. The final stool-culture result could not be independently verified from the available retrospective record and is therefore not described as definitively negative. Cytomegalovirus (CMV) immunoglobulin M was negative, and subsequent testing included quantitative CMV polymerase chain reaction (PCR) of peripheral blood and colonic biopsy tissue and CMV immunohistochemistry of the colonic biopsy, all of which were negative.

### 2.3 Radiological findings

Contrast-enhanced computed tomography (CT) of the chest, abdomen, and pelvis on 20 August 2025 demonstrated newly developed marked diffuse circumferential colonic wall thickening with submucosal edema involving the cecum, ascending colon, and proximal transverse colon, with marked pericolic fat stranding and mild free fluid; a short adjacent segment of sigmoid wall thickening was also noted (Figure 1). There was no pneumatosis intestinalis, portal venous gas, mesenteric vascular occlusion, pneumoperitoneum, abscess, or colonic mass lesion, and the liver and bone metastatic disease was unchanged from the July 2025 study. Because of the predominantly right-sided distribution, the initial interpretation raised concern for typhlitis, and the surgical team was consulted. As the patient was hemodynamically stable with no clinical evidence of peritonitis or perforation, conservative management was recommended: she was kept nil per os and treated with intravenous fluids, proton-pump inhibitor therapy, and intravenous ciprofloxacin and metronidazole. Surgical intervention was not indicated.

**FIGURE 1:**
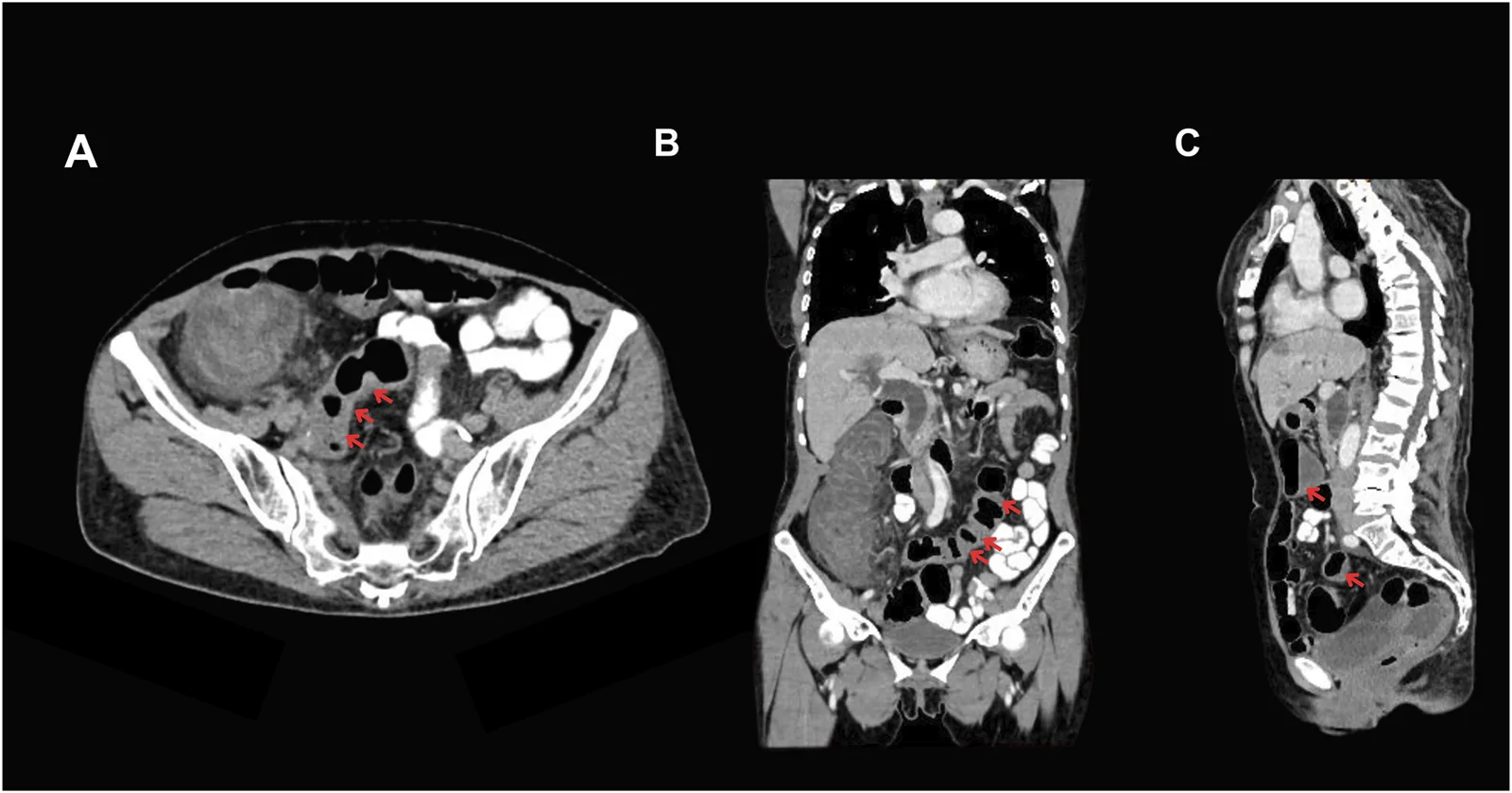
Contrast-enhanced computed tomography at presentation (20 August 2025). **(A)** Axial **(B)** coronal, and **(C)** sagittal images demonstrate marked circumferential wall thickening and submucosal edema of the cecum, ascending colon, and proximal transverse colon, with pericolic fat stranding. Red arrows indicate the principal affected segments and were added for illustration only; no diagnostic image content was altered.

### 2.4 Endoscopic and histopathological findings

Despite conservative treatment, diarrhea, abdominal cramps, and hematochezia persisted, and lower gastrointestinal endoscopy was performed at a referral gastroenterology center. Colonoscopy demonstrated diffuse mucosal erythema and edema throughout the colon, with an endoscopic impression of treatment-related colitis/mucositis; biopsies were obtained from the colon and terminal ileum. No ulceration, pseudomembranes, segmental demarcation with a sharply defined transition zone, mass lesion, or active arterial bleeding point was described in the endoscopy report. Representative endoscopic photographs could not be retrieved from the external referral center at which the procedure was performed and are therefore not included; the endoscopic component of this report relies on the documented procedure report and the corresponding biopsy specimens.

Histopathological examination of the colonic biopsy demonstrated moderate chronic active non-specific inflammation with focal surface epithelial erosion, preserved overall mucosal architecture, and a mixed inflammatory infiltrate in the lamina propria (Figure 2). No viral cytopathic changes were identified, CMV immunohistochemistry was negative, and there was no morphological evidence of inflammatory bowel disease, dysplasia, or malignancy. Specifically, there was no crypt distortion, basal plasmacytosis, granuloma, crypt apoptosis with crypt dropout, lamina propria hyalinization with withered crypts, or pseudomembrane formation. Terminal ileal biopsies showed no specific pathological abnormality and no evidence of microorganisms, dysplasia, or malignancy. Quantitative CMV PCR of colonic tissue and of peripheral blood was negative. The findings therefore confirmed active colonic mucosal inflammation and injury but were non-specific, and are interpreted further in Section 3.4.

**FIGURE 2:**
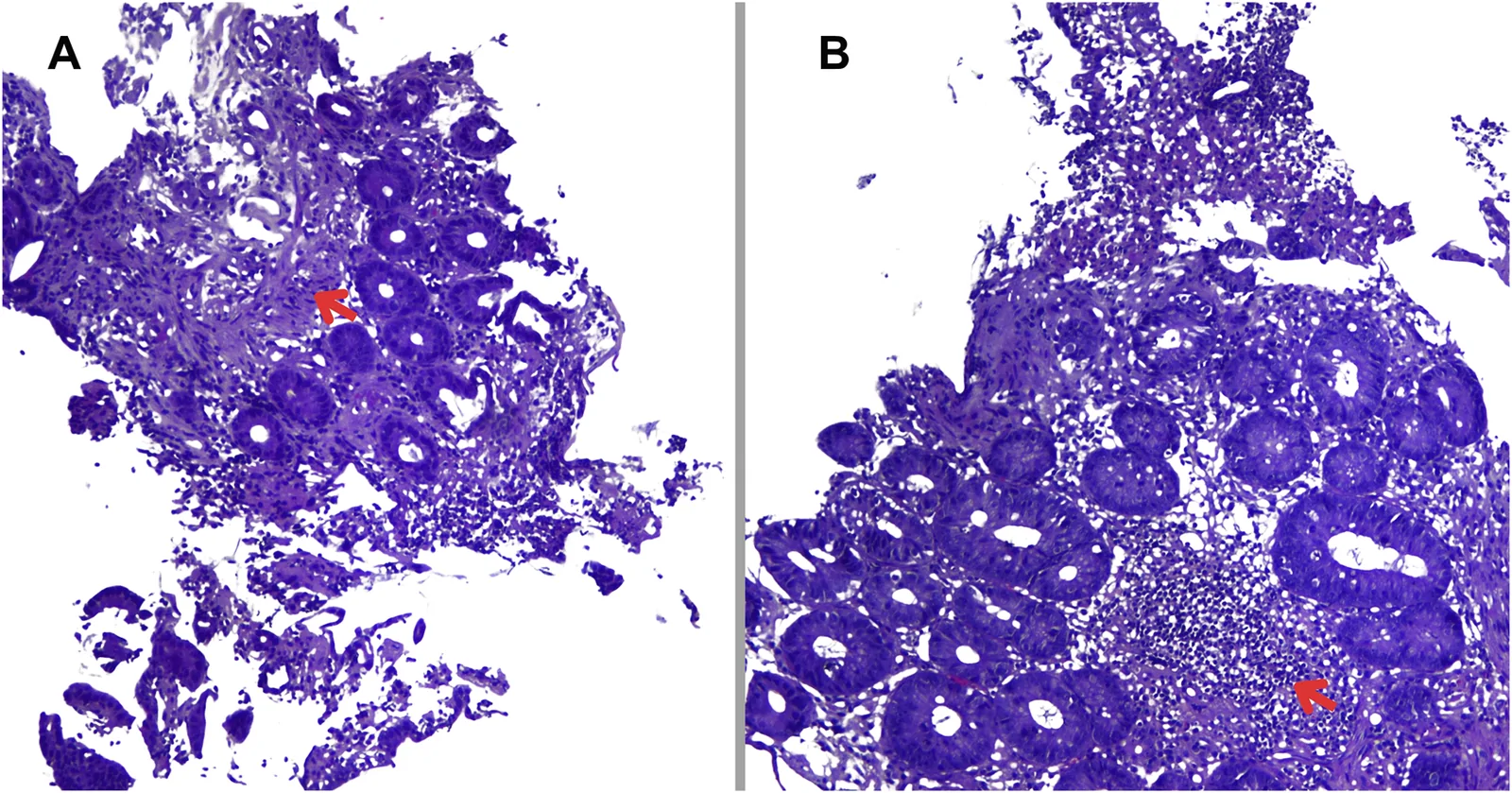
Colonic biopsy, hematoxylin and eosin. **(A,B)** Preserved crypt architecture with expansion of the lamina propria by a mixed inflammatory infiltrate (red arrows) and focal surface epithelial erosion, consistent with chronic active non-specific colitis. There were no viral cytopathic changes, crypt distortion, basal plasmacytosis, granulomata, pseudomembranes, dysplasia, or malignancy, and cytomegalovirus immunohistochemistry and tissue polymerase chain reaction were negative. The findings confirm active colitis but are non-specific and do not independently establish everolimus causality. Photomicrographs are reproduced as originally captured; no digital processing has been applied.

### 2.5 Diagnostic assessment and differential diagnosis

The differential diagnosis included infectious colitis, neutropenic enterocolitis, ischemic colitis, inflammatory bowel disease, malignant colonic involvement, radiation-related injury, anticoagulation-associated amplification of gastrointestinal bleeding, and drug-induced colitis.

Infectious colitis was considered less likely because the available stool investigations identified no infectious etiology, *C. difficile* toxin testing was negative, CMV PCR was negative in peripheral blood and colonic tissue, CMV immunohistochemistry was negative, and no viral cytopathic changes were identified histologically. CMV antigenemia was not separately performed, but CMV was investigated directly by quantitative PCR of blood and tissue and by tissue immunohistochemistry. Herpes simplex virus testing, stool ova-and-parasite microscopy, and *Cryptosporidium* enzyme immunoassay were not performed or could not be confirmed retrospectively; uncommon opportunistic infections therefore cannot be excluded with absolute certainty, a limitation carried into the causality assessment.

Neutropenic enterocolitis (typhlitis) was initially considered because of predominant cecal and right-hemicolonic involvement on CT, but the patient was not neutropenic, substantially reducing its likelihood. She had additionally not received cytotoxic chemotherapy since 2021, and the current regimen was an oral endocrine and mTOR-inhibitor combination not typically associated with severe mucosal cytotoxicity or profound neutropenia.

Ischemic colitis was considered given acute abdominal pain and hematochezia, but the patient remained hemodynamically stable, serum lactate was within the reference range, peritoneal signs were absent, and biopsy showed no characteristic ischemic pattern. The anatomical distribution was also atypical, involving predominantly the cecum and right hemicolon rather than the classical watershed distribution at the splenic flexure or rectosigmoid junction, and no mesenteric arterial occlusion or venous thrombosis was demonstrated on contrast-enhanced CT. A normal serum lactate does not independently exclude early or mucosa-limited ischemia; interpreted together with hemodynamic stability, absence of peritoneal signs, non-ischemic histology, and subsequent clinical evolution, the findings did not support advanced transmural ischemia or intestinal necrosis.

Inflammatory bowel disease was considered less likely given no known prior compatible history, preserved mucosal architecture, unremarkable terminal ileal biopsies, and no morphological features diagnostic of inflammatory bowel disease. The abrupt onset at the age of 60 in a patient with no antecedent gastrointestinal symptoms, and the complete and sustained resolution after drug withdrawal without maintenance immunosuppression, further argued against a newly presenting chronic idiopathic inflammatory bowel disease.

Malignant involvement was excluded, with no histopathological evidence of dysplasia or malignancy in the sampled colonic or terminal ileal tissue. CT demonstrated diffuse circumferential inflammatory wall thickening rather than a focal mass, shouldering, or obstructing lesion, and metastatic disease elsewhere was radiologically stable.

Radiation-related injury was considered unlikely because the dominant abnormalities were located in the cecum and right hemicolon, well outside the prior sacral radiation field, whereas radiation proctocolitis would be expected to involve the rectosigmoid within the treated volume.

Immune checkpoint inhibitor–associated colitis, an important consideration in any patient with cancer presenting with acute colitis, was not applicable, as the patient had never received an immune checkpoint inhibitor.

Anticoagulation with apixaban may have increased the clinical expression or severity of bleeding but could not explain the marked CT inflammatory abnormalities, diffuse endoscopic mucosal inflammation, or biopsy-confirmed chronic active colitis; apixaban was held during the acute bleeding episode.

Drug-induced colitis was considered the most likely diagnosis, on the grounds set out in Section 3.1.

### 2.6 Severity assessment

The adverse event was retrospectively graded using the National Cancer Institute CTCAE version 5.0 (National Cancer Institute, 2017) as Grade 3 colitis, on the basis of severe abdominal pain in the presence of objectively documented colitis and a medically significant course requiring hospitalization and substantial intervention: bowel rest, intravenous fluids, systemic corticosteroids, antimicrobial therapy during the diagnostic phase, correction of electrolyte abnormalities, blood transfusion during active hematochezia and symptomatic anemia, and subsequent total parenteral nutrition for persistent symptoms and inadequate oral intake.

Exact stool frequency above baseline was not reliably documented; a separate CTCAE diarrhea grade was therefore not assigned. Grade 4 criteria were not met, as there were no documented immediately life-threatening consequences, hemodynamic instability, bowel perforation, toxic megacolon, bowel necrosis, peritonitis requiring urgent intervention, emergency surgery, or intensive care unit admission; Grade 5 did not apply, as the event was not fatal. Because the proportion of anemia attributable specifically to acute colonic blood loss could not be quantified retrospectively, the transfusion requirement was not used as the sole basis for grading colitis or for independently assigning a gastrointestinal hemorrhage grade.

### 2.7 Formal causality assessment

A retrospective Naranjo Adverse Drug Reaction Probability Scale assessment yielded a score of 5, corresponding to a probable adverse drug reaction (Naranjo et al., 1981) (Table 1). The assessment supports probable rather than definite causality; the absence of rechallenge, lack of serum everolimus concentration measurements, concurrent therapeutic interventions, and incomplete testing for selected opportunistic infections preclude stronger causal attribution.

**TABLE 1:** Naranjo adverse drug reaction probability assessment.

Naranjo criterion	Case-specific assessment	Score
Previous conclusive reports of the reaction	Serious inflammatory, ulcerative, hemorrhagic, and perforating gastrointestinal toxicity has been reported with mTOR inhibitors	+1
Event appeared after administration of the suspected drug	Colitis developed after initiation of everolimus	+2
Reaction improved after withdrawal of the suspected drug	Clinical and radiological improvement followed permanent everolimus withdrawal and medical management	+1
Reaction appeared after rechallenge	Rechallenge was not performed	0
Alternative causes could have caused the reaction	Major alternatives were assessed, but some opportunistic infection testing was unavailable; scored conservatively as uncertain	0
Reaction reappeared with placebo	Not applicable	0
Drug detected in toxic concentration	Serum everolimus concentration was not measured	0
Dose-response relationship demonstrated	Not assessable	0
Similar reaction during previous exposure	No known previous similar reaction	0
Adverse event confirmed by objective evidence	CT, colonoscopy, and histopathology objectively confirmed colitis	+1
Total score	Probable adverse drug reaction	5

### 2.8 Treatment and clinical course

Everolimus was permanently discontinued. The patient received intravenous methylprednisolone 50 mg once daily, together with bowel rest, intravenous fluids, correction of electrolyte abnormalities, and completion of the intravenous ciprofloxacin and metronidazole course begun during evaluation for possible infectious colitis or typhlitis.

Two units of packed red blood cells were transfused for symptomatic anemia during ongoing hematochezia, after which hemoglobin improved to 11.4 g/dL. Follow-up CT on 8 September 2025 demonstrated complete resolution of the previously documented right-hemicolon inflammatory changes (Figure 3), with otherwise stable oncological findings on the radiological report. Despite radiological improvement, persistent gastrointestinal symptoms and inadequate oral intake required nutritional support, and total parenteral nutrition was started on 11 September 2025, followed by gradual clinical improvement including reduced diarrhea frequency. Gastroenterology consultation recommended postponing repeat lower endoscopy because of the recent acute mucosal inflammatory episode, gradual corticosteroid tapering, and continued proton-pump inhibitor therapy.

**FIGURE 3:**
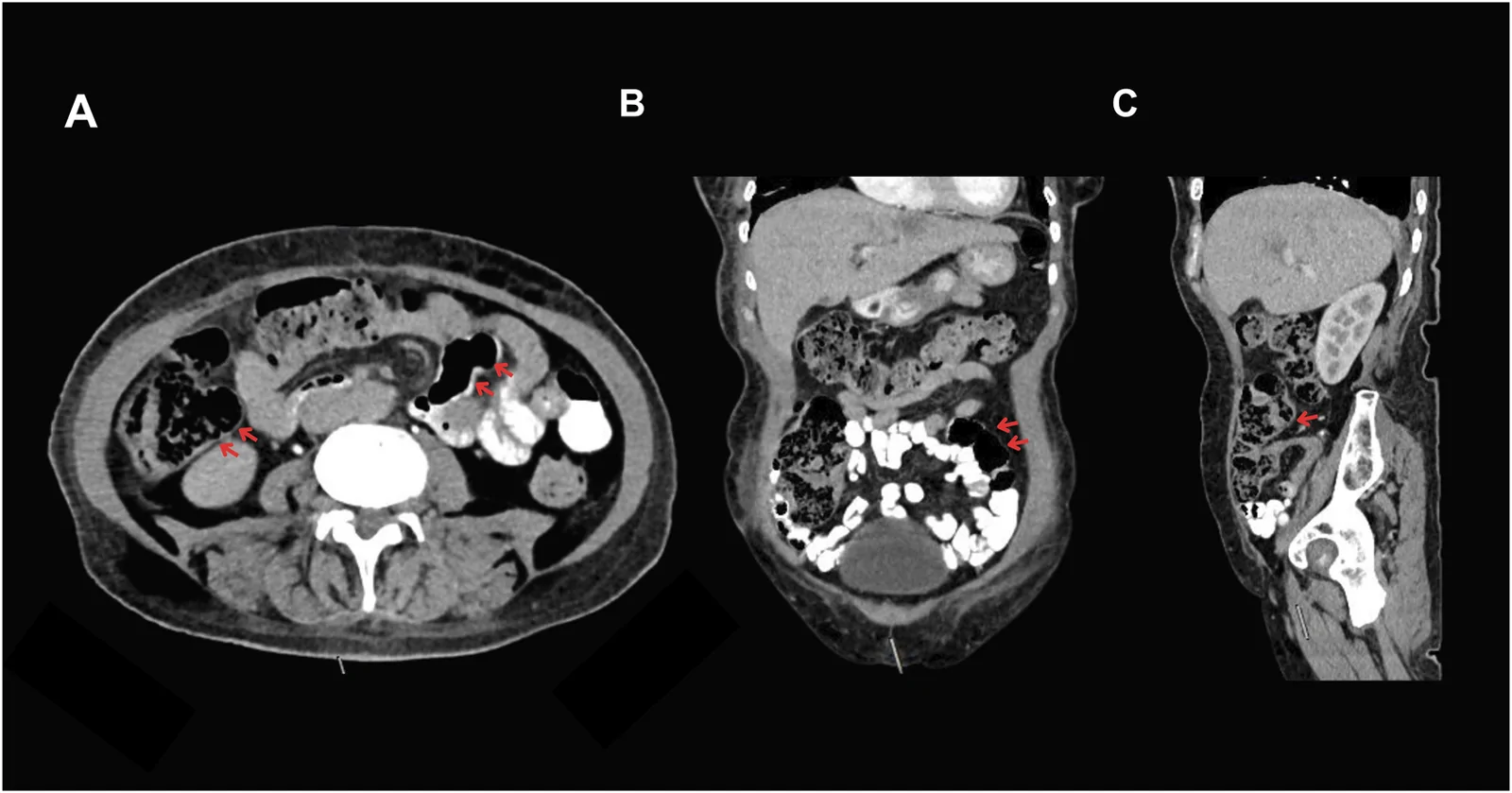
Follow-up contrast-enhanced computed tomography (8 September 2025). **(A)** Axial **(B)** coronal, and **(C)** sagittal images demonstrate normalization of colonic wall thickness with resolution of submucosal edema and pericolic inflammatory change. Red arrows were added for illustration only; no diagnostic image content was altered.

At subsequent follow-up, diarrhea and hematochezia had completely resolved, without recurrence of gastrointestinal symptoms. Everolimus remained permanently discontinued, and systemic anticancer treatment was continued with fulvestrant. During the available follow-up period, the patient remained alive, clinically stable, and free of recurrent gastrointestinal symptoms.

## 3 Discussion

We describe a case of probable everolimus-associated CTCAE Grade 3 severe colitis in a woman receiving everolimus for metastatic hormone receptor–positive, HER2-negative breast cancer. Approximately 6 months after starting the everolimus-containing regimen, she developed acute severe abdominal pain, bloody diarrhea, and hematochezia, with complementary objective evidence of colitis on CT, colonoscopy, and histopathology, a negative available infectious evaluation, and radiological and then complete clinical resolution after permanent everolimus withdrawal and medical management.

### 3.1 Diagnostic reasoning and attribution to everolimus

Drug-associated colitis is largely a clinicopathological diagnosis. No single laboratory test, imaging characteristic, endoscopic appearance, or histopathological pattern is usually sufficient to establish causality, which instead requires integration of drug exposure, latency, clinical phenotype, objective evidence of inflammation, consideration of competing diagnoses, and evolution after drug withdrawal.

Applying that standard here, infectious causes were addressed most thoroughly for CMV, which was assessed by three complementary direct methods, all negative and without viral cytopathic changes. The evaluation was nonetheless not exhaustive, as herpes simplex virus, stool ova-and-parasite microscopy, and *Cryptosporidium* testing were unavailable, so no claim of complete infectious exclusion is made. The remaining alternatives were each addressed in turn (Section 2.5) and none was supported by the available evidence. Integrating the temporal association with everolimus, the multimodal evidence of colitis, the absence of a more persuasive alternative, radiological and clinical resolution after permanent withdrawal, and the lack of recurrence, a probable association with everolimus was supported, with the Naranjo score providing a formal framework and categorizing the reaction as probable rather than definite.

### 3.2 A practical framework for the differential diagnosis of acute colitis in the patient receiving systemic anticancer therapy

Because the reasoning applied in this case has broader teaching value, we set it out here as a general framework. Acute colitis in a patient on systemic anticancer therapy is not a single entity but a convergence point for at least eight mechanistically distinct processes, several of which may coexist. The clinical, radiological, endoscopic and histological features that discriminate between them are summarized below.

Infectious colitis remains the first consideration in every case, because it is common, treatable, and because immunosuppressive therapy for a presumed drug reaction may be actively harmful if an infection is missed. *Clostridioides difficile* infection is suggested by recent antibiotic or proton-pump inhibitor exposure, prior hospitalization and, endoscopically, by pseudomembranes, although these are absent in a substantial minority; testing should follow a two-step algorithm combining a sensitive screening assay with a toxin assay. CMV colitis is disproportionately important in patients with malignancy, prior corticosteroid exposure or lymphopenia, and characteristically produces deep, well-demarcated ulcers with viral inclusion bodies and positive immunohistochemistry. Because serology and blood PCR may be negative in tissue-limited disease, direct tissue interrogation by immunohistochemistry and tissue PCR is the more reliable approach. Bacterial enteropathogens, parasitic infection and, in the profoundly immunosuppressed, *Cryptosporidium*, *Isospora*, *Microsporidia* and herpes simplex virus should be sought where the epidemiological context or degree of immunosuppression warrants it. A minimum acceptable evaluation comprises stool culture, *C. difficile* testing, CMV assessment of tissue and, where feasible, a multiplex gastrointestinal PCR panel and ova-and-parasite microscopy.

Neutropenic enterocolitis (typhlitis) should be considered whenever cecal and right-hemicolonic wall thickening is present, but it is essentially a diagnosis of the neutropenic patient, classically 10–14 days after intensive cytotoxic chemotherapy. Absolute neutrophil count is therefore the single most discriminating variable. Marked mural thickening (frequently >4 mm), pneumatosis, and a fulminant septic course support the diagnosis. In its absence, and particularly where the regimen is an oral endocrine or targeted agent rather than a myelosuppressive cytotoxic, alternative explanations for right-sided colitis should be pursued rather than assumed.

Ischemic colitis shares the phenotype of acute abdominal pain followed by hematochezia and is therefore a persistent mimic. Discrimination rests on distribution and evolution rather than on any single test: ischemia preferentially affects the watershed territories at the splenic flexure and rectosigmoid junction, produces a segmental abnormality with an abrupt transition to normal mucosa endoscopically, and yields a characteristic histology of lamina propria hyalinization, withered or atrophic crypts and coagulative mucosal necrosis. Serum lactate is insensitive to mucosa-limited ischemia and a normal value does not exclude it, a point that deserves emphasis because normal lactate is frequently over-interpreted as reassuring. Where the distribution is right-sided, the histology non-ischemic, and no vascular occlusion is demonstrated, ischemia becomes substantially less likely.

Immune checkpoint inhibitor–associated colitis is now among the most frequent causes of drug-induced colitis in oncology and must be actively excluded by medication review. It typically arises within weeks to a few months of initiation, may involve the colon diffusely or segmentally, and histologically shows neutrophilic cryptitis with crypt abscesses and increased crypt apoptosis. Neither the endoscopic nor the histological appearance reliably distinguishes checkpoint-inhibitor colitis from other drug-induced colitides; the discriminator is exposure history.

Chemotherapy- and targeted-agent–associated mucosal injury other than checkpoint inhibition includes fluoropyrimidine and irinotecan enterocolitis, taxane-associated colitis, tyrosine kinase inhibitor–related diarrhea, and mTOR inhibitor–associated inflammatory injury. These generally produce non-specific active colitis without a pathognomonic pattern, which is precisely why attribution depends on temporality, dechallenge and the systematic elimination of alternatives rather than on biopsy.

Idiopathic inflammatory bowel disease presenting *de novo* is uncommon but not negligible in later life, given the recognized second incidence peak in the sixth and seventh decades. Chronicity markers on biopsy, namely crypt architectural distortion, basal plasmacytosis, Paneth cell metaplasia and granulomata, are the key discriminators. Their absence, together with an abrupt onset, no antecedent gastrointestinal history and complete resolution after withdrawal of a suspected drug without maintenance therapy, argues strongly against it.

Radiation colitis or proctitis is defined by anatomy: the abnormality must lie within the treated volume. Late radiation injury may present months to years after treatment with telangiectasia, mucosal pallor and friability, and submucosal fibrosis histologically. A distribution that does not correspond to the radiation field effectively excludes it.

Direct malignant involvement of the colon, whether by metastasis, peritoneal deposits or a synchronous primary, should be considered in any patient with known disseminated malignancy. It is addressed by targeted biopsy of any focal lesion together with careful review of cross-sectional imaging for a mass, shouldering, or obstructive features rather than diffuse circumferential inflammatory thickening.

Finally, anticoagulant and antiplatelet therapy deserves separate consideration. Anticoagulation does not cause colitis, but it markedly modifies its clinical expression: a mucosal inflammatory process that would otherwise produce diarrhea alone may present with frank hematochezia and transfusion-requiring anemia. Recognizing anticoagulation as an amplifier of the bleeding phenotype rather than as the primary cause avoids two opposite errors: attributing the entire syndrome to the anticoagulant and thereby missing the underlying colitis, or ignoring its contribution when grading severity.

Applied prospectively, this framework suggests a pragmatic sequence. Quantify the neutrophil count and hemodynamic status first, as these determine whether typhlitis and transmural ischemia are in play and whether surgical consultation is urgent. Obtain contrast-enhanced CT to define distribution and exclude perforation, pneumatosis and mass lesion. Send a full infectious panel including *C. difficile* before initiating corticosteroids. Perform lower endoscopy with biopsies from both involved and uninvolved mucosa, including CMV immunohistochemistry and tissue PCR, once the acute risk of perforation is judged acceptable. Finally, conduct a systematic medication review encompassing checkpoint inhibitors, cytotoxics, targeted agents, non-steroidal anti-inflammatory drugs, antibiotics and anticoagulants. Only when this sequence has been completed does the attribution of a non-specific active colitis to a specific drug become defensible, and even then it remains a probabilistic rather than a definitive judgment.

### 3.3 Objective basis for the severe (Grade 3) classification

The Grade 3 classification reflects severe abdominal pain in the presence of objectively documented colitis and a medically significant course requiring hospitalization and substantial management, rather than any single feature. The term “severe colitis” is used here to denote a Grade 3, medically significant adverse event and should not be interpreted as synonymous with Grade 4 life-threatening toxicity, the criteria for which were not met.

### 3.4 Histopathology and its relationship to gastrointestinal bleeding

The histopathological findings were clinically important but non-specific: moderate chronic active inflammation with a mixed lamina propria infiltrate, focal surface epithelial erosion, and preserved architecture confirmed active mucosal injury without a drug-specific pattern. Their greatest value was in the assessment of competing diagnoses. The absence of a prominent hemorrhagic pattern in the selected fragments does not contradict the documented bloody diarrhea and hematochezia, because endoscopic forceps biopsies sample small areas of a large, potentially heterogeneous mucosal surface, and bleeding can be focal or intermittent and may originate from areas not represented in the specimen. Histopathology alone could therefore establish neither the anatomical source of bleeding nor everolimus as the specific cause. The most accurate pathological diagnosis is chronic active non-specific colitis with focal surface epithelial erosion; attribution to everolimus depends on clinicopathological correlation and the complete diagnostic sequence rather than on a pathognomonic histological signature.

### 3.5 Gastrointestinal toxicity of mTOR inhibitors in the literature

Severe inflammatory and hemorrhagic gastrointestinal toxicity from mTOR inhibition appears uncommon but is clinically plausible. Arakawa et al. reported everolimus-associated hemorrhagic colitis in a patient with a nonfunctional pancreatic neuroendocrine neoplasm, with inflammatory and bleeding lesions endoscopically, non-specific inflammatory histology, and improvement after withdrawal (Arakawa et al., 2022). Tsunematsu et al. reported severe everolimus-related gastrointestinal hemorrhage (Tsunematsu et al., 2019), and Abe et al. described multiple small-intestinal ulcers with perforation during everolimus treatment for a rectal neuroendocrine tumor, extending the spectrum to ulcerative and perforating injury (Abe et al., 2023). Severe intestinal injury has also been reported in a patient with metastatic breast cancer receiving everolimus, adding relevance to this population (Song et al., 2020). Enteritis has been described with both everolimus and temsirolimus, raising the possibility of a rare class-related effect of mTOR pathway inhibition (Parithivel et al., 2011); however, the small number of published cases, heterogeneity of presentation, differing populations, and non-specific pathology prevent definition of a uniform phenotype. Across these reports the latency from initiation to gastrointestinal event has varied from weeks to many months, the anatomical site has ranged from small intestine to colon, and the histology has been consistently non-specific, a pattern that reinforces the conclusion that this toxicity cannot presently be diagnosed by any single modality and must instead be recognized clinically. The present case adds a patient treated for metastatic hormone receptor–positive breast cancer who presented with CTCAE Grade 3 colitis and hematochezia, documented across three modalities, with complementary direct CMV testing and sustained recovery after permanent discontinuation.

### 3.6 Potential pathophysiological mechanisms

The mechanism of severe everolimus-associated gastrointestinal injury has not been established. Because mTOR signaling participates in epithelial proliferation, cellular survival, metabolic regulation, immune signaling, angiogenesis, and tissue repair, mTOR inhibition could plausibly impair epithelial regeneration and mucosal healing, alter local immune homeostasis, or increase microvascular vulnerability. Prior reports have proposed disruption of mTOR-dependent epithelial repair or angiogenic signaling as predisposing susceptible patients to mucosal injury, ulceration, or bleeding, but these remain hypotheses and were not directly demonstrated here; the heterogeneity of reported toxicities further argues against a single established mechanism. The mechanistic discussion should therefore be regarded as biologically plausible but speculative: the case provides clinical evidence of an association, not a molecular mechanism.

### 3.7 Management and outcome

The principal intervention was permanent withdrawal of everolimus, alongside bowel rest, intravenous fluids, electrolyte replacement, initial antimicrobial therapy, systemic corticosteroids, blood transfusion for symptomatic anemia, and nutritional support including total parenteral nutrition. Because these were administered concurrently, recovery cannot be attributed to corticosteroid therapy alone; it is most accurately interpreted as following permanent everolimus withdrawal together with comprehensive medical and supportive management. Notably, radiological resolution preceded complete symptomatic recovery, with CT normalizing while persistent symptoms and inadequate oral intake still required total parenteral nutrition. No rechallenge was performed: although a positive rechallenge could theoretically strengthen causal attribution, deliberate re-exposure would have been difficult to justify after a severe Grade 3 gastrointestinal adverse event with clinically significant bleeding.

### 3.8 Limitations

This case report has several limitations. Causality is probable rather than definitive, with no specific biomarker or pathognomonic histopathological finding establishing everolimus as the cause, and the histopathological findings cannot independently distinguish everolimus-associated injury from other causes of active colitis. No everolimus rechallenge was performed, which limits causal certainty. Representative endoscopic photographs could not be retrieved from the external referral center where the colonoscopy was performed, so the endoscopic component relies on the documented procedure report and the accompanying biopsy specimens; no endoscopic images are presented in this report. Although substantial, the infectious evaluation did not include herpes simplex virus, stool ova and parasites, or *Cryptosporidium*, so rare infectious causes cannot be excluded with absolute certainty, and the final stool-culture result could not be independently verified from the available retrospective record. Exact daily stool frequency above baseline was unavailable, so severity grading relies on CTCAE colitis criteria and the overall medically significant course rather than stool-frequency criteria. Serum everolimus concentrations were not measured, precluding assessment of any exposure–toxicity relationship. Concomitant apixaban may have amplified the gastrointestinal bleeding phenotype, although it cannot explain the objectively documented inflammatory colitis. Repeat colonoscopy was not performed after recovery, so endoscopic and histological resolution was not confirmed; documentation of recovery rests on cross-sectional imaging and sustained clinical remission. Finally, because multiple treatments were administered concurrently, the individual contributions of everolimus withdrawal, corticosteroid therapy, antimicrobial treatment, bowel rest, and supportive management to recovery cannot be separated.

Despite these limitations, the temporal association, multimodal objective evidence of colitis, negative available infectious investigations, absence of a more persuasive competing diagnosis, radiological and clinical recovery following permanent everolimus withdrawal, absence of recurrence during available follow-up, and Naranjo score of five collectively support a probable causal association.

## 4 Conclusion

This case describes probable everolimus-associated CTCAE Grade 3 severe colitis in a patient with metastatic hormone receptor–positive, HER2-negative breast cancer who developed severe abdominal pain, bloody diarrhea, and hematochezia, with radiological, endoscopic, and histopathological evidence of active colitis. The histopathological findings were compatible with the overall presentation but were not specific for everolimus-associated injury and did not independently establish causality; the diagnosis rested instead on the integration of exposure, objective evidence, structured exclusion of alternatives, and clinical evolution after withdrawal, formalized by a Naranjo assessment indicating a probable adverse drug reaction.

Clinicians should consider mTOR inhibitor–associated gastrointestinal toxicity when patients receiving everolimus develop acute abdominal pain, significant diarrhea, hematochezia, or other evidence of gastrointestinal bleeding. A structured differential diagnosis of the kind presented here, undertaken before immunosuppressive therapy is initiated, together with early recognition, appropriate withdrawal of the suspected causative agent, and multidisciplinary management, may facilitate recovery, prevent progression to more serious complications, and avoid unnecessary surgical intervention.

## Data Availability

The original contributions presented in the study are included in the article/supplementary material, further inquiries can be directed to the corresponding author.
